# Editorial: Pseudocereals as sustainable alternative crops for food production amid ongoing climate change

**DOI:** 10.3389/fpls.2026.1870319

**Published:** 2026-05-19

**Authors:** Henda Mahmoudi, Sajad Ali, Ahmed Debez, Nieves Aparicio

**Affiliations:** 1 Crops Diversification & Genetics (CDG) Section, International Center for Biosaline Agriculture (ICBA), Dubai, United Arab Emirates; 2 Department of Biotechnology, College of Life and Applied Sciences, Yeungnam University, Gyeongsan, Republic of Korea; 3 Laboratoire des Plantes Extremophiles (LPE), Center of Biotechnology of Borj Cedria (CBBC), Hammam-Lif, Tunisia; 4 Subdirección de Investigación y Tecnología, Instituto Tecnológico Agrario de Castilla y León, Valladolid, Spain

**Keywords:** amaranth, buckwheat, climate change, food security, pseudocereals, quinoa, sustainable agriculture

## Impact statement

In a rapidly warming world, pseudocereals offer a realistic and scalable pathway to strengthen climate resilience, improve nutritional outcomes, and support sustainable agricultural transformation across vulnerable regions.

Climate change is no longer a distant projection; it is a present and accelerating reality that is reshaping global agriculture. Rising temperatures, erratic rainfall, increasing soil salinity, and more frequent extreme weather events are placing unprecedented pressure on crop productivity and food systems worldwide. For decades, global food security has depended heavily on a small number of staple cereals. However, this reliance is becoming increasingly fragile under climate stress. In response, there is a growing recognition of the need to diversify cropping systems with resilient, nutrient-dense alternatives.

Pseudocereals, particularly quinoa (*Chenopodium quinoa*), amaranth (*Amaranthus* spp.), and buckwheat (*Fagopyrum* spp.), have emerged as promising candidates in this transition. These crops combine remarkable tolerance to abiotic stress with high nutritional value, making them uniquely suited to address both environmental and dietary challenges. This Research Topic brings together seven contributions that collectively explore the physiological resilience, agronomic potential, genetic diversity, and broader relevance of pseudocereals under ongoing climate change.

One of the defining strengths of pseudocereals lies in their physiological adaptability. The study on quinoa leaf responses to high temperatures (Fishbein et al.) demonstrates that quinoa can maintain photosynthetic performance under elevated temperatures, activating protective mechanisms under moderate stress. Such findings highlight the intrinsic resilience of pseudocereals and their potential to sustain productivity where traditional crops may fail.

At a broader scale, the question of whether pseudocereals can complement or even replace conventional cereals is addressed in the review (Sun et al.). This work underscores quinoa’s adaptability to saline and marginal environments, reinforcing its relevance as a future-ready crop in the face of environmental degradation.

The importance of pseudocereals is particularly evident in arid and semi-arid regions. The review on pseudocereals in arid environments (Manoharan et al.) emphasizes their capacity to thrive under water scarcity and poor soil conditions. Similarly, the comprehensive review on amaranth (Macías-Naranjo et al.) revisits this ancient crop, highlighting both its resilience and the need for renewed scientific and agronomic attention.

In addition to resilience, improving productivity and farming practicality is essential. The dual-purpose quinoa harvesting approach (Ndunguru et al.) offers a compelling example of innovation, allowing farmers to obtain both leafy biomass and grain from a single crop cycle. This approach not only enhances productivity but also improves resource efficiency, which is critical for smallholder systems.

Advances in genetics further strengthen the case for pseudocereals. The genome-wide analysis of buckwheat (Manzoor et al.) identifies key markers associated with nutraceutical traits, paving the way for targeted breeding of nutrient-rich varieties. Such molecular insights are essential for aligning crop improvement with nutritional security goals.

Finally, while this Research Topic focuses on pseudocereals, it also situates them within a broader context of climate-resilient crops. The review on millets (Mukherjee et al.) reinforces the importance of diversification beyond conventional staples, highlighting parallel strategies for building resilient food systems.

Together, these contributions paint a coherent picture: pseudocereals are not merely alternative crops, they are strategic assets for the future of agriculture. However, their widespread adoption will depend on overcoming key barriers, including limited seed systems, underdeveloped markets, and low consumer awareness. Addressing these challenges will require coordinated efforts across research, policy, and value chains.

In conclusion, pseudocereals offer a compelling combination of resilience, nutrition, and adaptability. In a world where climate uncertainty is becoming the norm, these crops provide not just an alternative, but a necessary evolution in how we produce food. The work presented in this Research Topic represents an important step toward unlocking their full potential and integrating them into sustainable, climate-smart agricultural systems.

